# Within-farm transmission characteristics of bluetongue virus serotype 8 in cattle and sheep in the Netherlands, 2007-2008

**DOI:** 10.1371/journal.pone.0246565

**Published:** 2021-02-08

**Authors:** Thomas J. Hagenaars, Anoek Backx, Eugene M. A. van Rooij, Roger M. M. I. Vrouenraets, Daniel M. Bontje, Annemarie Bouma, Armin R. W. Elbers

**Affiliations:** 1 Wageningen Bioveterinary Research, Lelystad, The Netherlands; 2 DAC Heerlen, Heerlen, The Netherlands; 3 Ministry of Agriculture, Nature and Food Quality, The Hague, The Netherlands; Faculty of Science, Ain Shams University (ASU), EGYPT

## Abstract

In 2006 and 2007, sheep and cattle farms in the Netherlands were affected by an epidemic of bluetongue virus serotype 8 (BTV-8). In order to obtain insight into the within-farm spread of the virus, five affected cattle and five affected sheep farms were longitudinally monitored between early 2007 and mid or late 2008. The farms were visited between four and seven times to collect blood samples. During each visit, all animals present in the flock or herd were sampled. The samples were analysed for the presence of BTV-8 antibodies (ELISA) and BTV-8 antigen (rRT-PCR). The observed patterns of RT-PCR positives indicate a rapid within-farm virus spread during the vector season. During vector-free periods we observed a complete rRT-PCR positivity decline within a few months. During the vector season a lower bound estimate of the basic reproduction number (R_0_) ranges from 2.9–6.9 in the cattle herds (one herd not analysed), and from 1.3–3.2 in the sheep flocks in this study.

## Introduction

Bluetongue (BT) is a non-contagious, *Culicoides*-borne disease of wild and domestic ruminants, caused by bluetongue virus (BTV), which is an orbivirus belonging to the family of *Reoviridae* [1]. The sequential occurrence of BT epidemics in the USA, the Iberian Peninsula, Middle East, Asia and southern Europe in the second half of the 20th century, led to the inclusion in 1963 of BT in the former list A of the World Organization for Animal Health (OIE) [2], due to the impact outbreaks may cause to the international trade of animals and animal products.

Prior to 1998, occasional incursions of BTV occurred in the southern part of Europe (Spain, Portugal, Greece and Cyprus) [3], but since 1998 several different BTV strains have invaded Europe [4] and an endemically infected situation now persists in southern European countries. BTV serotype 8 (BTV-8) emerged in The Netherlands, Belgium, France and Germany for the first time in 2006 and in the following year it spread rapidly throughout the rest of north-western Europe [5, 6]. A coordinated transnational vaccination campaign promoted by the European Commission started in the spring of 2008, with the expectation that the epidemic could be controlled by an effective use of vaccination. After BTV-8 was not detected in Europe for several years since 2010, European countries obtained an OIE ‘self-declared BTV-free’ status in 2012 and 2013 [7]. Surveillance programmes were implemented in the Member States of the EU in line with European legislation (Commission Regulation EC/1266/2007) with the aim to detect the possible virus circulation in a bluetongue-free Member State.

Despite being undetected in Europe for 5 years, BTV-8 re-emerged in August 2015 in the central part of France and spread throughout the country in 2016 [8], continuing its expansion in 2017 and 2018 [9]. Due to ongoing circulation of the virus in France, neighbouring countries (Germany, Switzerland, and Belgium) experienced BTV-8 introductions in the period 2017–2018 [10–12]. The BTV-8 serotype is considered special in comparison to other serotypes, notably in its ability to cause serious disease in cattle and goats, and the possibility of transplacental transmission [13].

In order to understand the epidemiology of vector-borne diseases including BT, to examine the potential spread of epidemics, and to assess the impact of control measures such as vaccination and animal movement control, mathematical transmission models have been developed [14–17]. Models for within-farm transmission often consider both the ruminant host species and the *Culicoides* (midge) vector [16]. Critical parameters in such a transmission model are the vector-to-host ratio, the duration of viremia in the ruminant host, the extrinsic incubation period of the virus in the vector, the biting rate, and probabilities of transmission per bite [14]. A less detailed within-farm transmission modelling approach leaves the vector implicit, and explicitly considers only the ruminant host, distinguishing Susceptible (i.e. uninfected), Infected and Recovered (SIR) hosts [18]. With the availability of suitable field information (infection status over time, based on antibody detection by ELISA and antigen detection by polymerase chain reaction (PCR)) in the ruminant hosts, the latter type of modelling may allow for the estimation of the within-farm reproduction number of BTV between ruminant hosts. In our study, shortly after the start of the BTV-8 epidemic in 2006, five dairy cattle herds and five sheep flocks in the southern part of the Netherlands were monitored longitudinally by whole-farm blood sampling, for detection of antibodies and BT virus. We first discuss the prevalence patterns arising from this unique field information, providing detailed information on the time evolution of the within-herd and within-flock spread of BTV-8. Subsequently we quantify this spread by using a SIR-type modelling approach to estimate the within-farm basic reproduction number.

## Materials and methods

### Study population

The study started in December 2006, after the first outbreaks had occurred in the geographic centre of the epidemic in the most southern part of the Netherlands (province of Limburg). A serological survey of cattle in the winter period of 2006–2007 indicated that BTV-8 had spread both within- and between cattle farms in the province of Limburg [19]. In 2006, a total of 22,301 dairy farms and 14,071 sheep farms were present in the Netherlands, of which 695 (3.1%) dairy farms and 423 (3.0%) sheep farms in the province of Limburg (Tables 41c and 43c in [20]). For practical and cost reasons, in total 5 cattle herds and 5 sheep flocks were selected for a longitudinal study, situated in the province of Limburg. The selection was based on farmers’ willingness to cooperate.

### Sampling

The study period spanned two winter periods without midge activity (vector-free period) and one summer in between with midge activity (vector season). During the study period there were four to seven visits to the herds or flocks in which blood samples were taken from all animals present; these visits were timed such as to obtain information on changes in prevalence during both the vector season and the vector-free period. Samples were labelled with individual animal identification number. The first visit was on December 28^th^, 2006, (cattle herd 5) and the last on July 10^th^, 2008, (cattle herd 3); for a complete list of sampling dates we refer to (Tables 1 and 2). According to official estimates of the Dutch Food and Consumer Product Safety Authority (NVWA) the vector-free period in the first winter lasted from 30 December 2006 until 27 March 2007 and the second vector-free period from 12 December 2007 until 22 April 2008. In the present study, no information was recorded on the age of the sheep animals. In two of the cattle herds, information on the age of the animals present was recorded at the first sampling date. The mean age of the animals in these herds was 3 years in both herds and the age ranges were 0–9 and 0–13 years.

**Table 1:** Overview of observations in cattle herds.

Date	# animals tested by serology	# seropositive	# animals tested by PCR	# PCR positive	# sero- and/or PCR positive
Herd 1					
10-01-2007	104	41	104	30	41
29-03-2007	107	40	107	3	40
15-11-2007	103	96	103	29	96
31-01-2008	112	106	112	7	106
07-04-2008	114	103	114	0	103
Herd 2					
12-01-2007	82	60	82	27	61
21-03-2007	83	60	83	3	60
07-06-2007	50	37	50	0	37
18-11-2007	71	69	71	13	69
19-03-2008	89	84	90	1	84
Herd 3					
21-01-2007	43	33	43	23	33
30-03-2007	41	32	41	1	32
25-10-2007	15	15	14	1	15
11-12-2007	19	19	19	0	19
10-07-2008	13	13	13	0	13
Herd 4					
16-01-2007	26	9	26	6	9
27-04-2007	29	11	29	0	11
04-10-2007	29	25	29	9	25
31-01-2008	21	17	21	3	17
Herd 5					
28-12-2006	111	69	112	60	70
01-03-2007	106	63	106	15	64
10-04-2007	106	64	106	3	64
08-06-2007	67	38	67	0	38
09-08-2007	62	43	62	0	43
15-12-2007	101	83	101	23	83
21-03-2008	97	73	97	0	73

Shaded rows: sampling dates within vector-free periods as defined by the official estimates of the Dutch Food Safety Authority (NVWA) (vector-free periods lasting from 30 December 2006 until 27 March 2007 and from 12 December 2007 until 22 April 2008).

**Table 2:** Overview of observations in sheep flocks.

Date	# animals tested by serology	# seropositive	# animals tested by PCR	# PCR positive	# sero- and/or PCR positive
Flock 1					
12-01-2007	118	16	118	8	16
02-03-2007	88	15	88	3	15
31-08-2007	115	66	115	48	73
18-11-2007	90	72	90	38	72
23-04-2008	77	62	77	0	62
Flock 2					
28-12-2006	14	0	14	0	0
11-04-2007	27	0	27	0	0
22-08-2007	22	14	22	15	17
26-10-2007	21	20	21	20	20
18-12-2007	13	13	13	11	13
07-04-2008	12	12	12	0	12
Flock 3					
21-01-2007	36	1	36	1	1
30-03-2007	15	0	15	0	0
13-07-2007	15	0	15	0	0
21-08-2007	14	6	14	4	8
Flock 4					
29-01-2007	86	4	86	0	4
08-03-2007	87	5	87	0	5
24-09-2007	79	48	79	27	48
02-01-2008	77	52	77	28	54
15-04-2008	44	35	44	0	35
Flock 5					
25-01-2007	297	1	297	0	1
18-05-2007	293	1	293	0	1
18-09-2007	432	144	432	144	166
21-02-2008	303	97	303	47	97
05-05-2008	262	79	261	0	79

Shaded rows: sampling dates within vector-free periods as defined by the Dutch Food and Product Safety Authority (NVWA) (vector-free periods lasting from 30 December 2006 until 27 March 2007 and from 12 December 2007 until 22 April 2008).

In Flock 5, part of the second round of sampling was performed on 13 April 2007, part on 18 May 2007.

### Diagnostic testing

Whole (EDTA) blood was collected for the detection of virus by an in-house developed real-time reverse transcriptase PCR (rRT-PCR) detecting all 24 traditional serotypes of BTV [21]. From the EDTA blood, isolated dsRNA samples were tested using primers (Eurogentec Nederland b.v., Maastricht, Netherlands), the forward primer is 5’-AGTGTCGCTGCCATGCTATC-3’ and the reverse primer is 5’-GCGTACGATGCGAATGCA-3’, and a Taqman probe 5’-6FAM-CGAACCTTTGGATCAGCCCGGA-XTMR-PH (Tib MolBiol, Berlin, Germany) targeting segment 10 of the BTV genome. RRT-PCR was performed by use of the LightCycler RNA Master Hybridization Probes kit (Kit, Roche Diagnostics Nederland b.v., Almere, Netherlands) with a LightCycler 2.0 (Roche Diagnostics Nederland BV, Almere, Netherlands). Three positive controls were included containing different virus dilutions of BTV1 grown on BHK21 cells in DMEM with 5% fetal bovine serum and diluted in the same growth medium. A run was successful when all negative controls were negative, and positive controls were positive. Results of test samples were considered positive if the software generated a crossing point (cp) value and a sigmoid curve with a signal (OD530/OD640) at least partly above the cut-off value. Results were considered doubtful in case of a sigmoid-shaped curve completely below the cut-off value, or in case of all other not interpretable curves.

Furthermore, the sera were tested for BTV specific antibodies in a commercial competitive ELISA (ID Screen® bluetongue Competition ELISA kit, ID Vet, Montpellier, France) according to manufacturer’s instructions. Results were measured as binary variable: positive or negative.

The test results, i.e. the data on which our analyses were based, are available as S1 File.

### Description of prevalence patterns

We summarized the test results by listing the total number of animals tested, the numbers of animals found seropositive and the numbers of animals found (rRT-)PCR positive at each sampling time point. Taking PCR positivity as a proxy for being infectious, the latter numbers describe the pattern of apparent infection prevalence. As the time between samplings was several months, the numbers of animals born or moved off the farm between consecutive observations were non-negligible. For this reason we also listed the number of between-samplings status conversions amongst animals present at both consecutive samplings, e.g. the number of negatives turning positive and vice versa.

### Estimation of transmission parameters

Leaving the role of the midges implicit, we adopted a simple SIR-type description of transmission during the vector season and use it to estimate a minimum value for the net between-ruminant basic reproduction number *R*_0_. A number of methods are available to estimate *R*_0_ based on such an SIR description, although we should note that none of these methods was designed for a situation in which non-negligible numbers of animals are born or moved off the population between consecutive observations. Established methods for the case where temporal information on the infection status of all individuals in the population have been obtained, are the methods designed for analysing small-scale transmission experiments: the final-size method [22] and the ‘generalized linear model’ (GLM) analysis (see e.g. [23]). In our study however, it turned out that the population sizes were too large to apply the final-size method. Furthermore, as will become clear in the results, between the most interesting consecutive sampling points in our data, infection status changes occurred for a large proportion of the population, and this prevented meaningful application of the GLM analysis. We therefore used the simpler approach of applying the final-size equation [24] to the field data; in contrast to the final-size method this approach yields only point estimates and no confidence bounds. More specifically, we used the version of this equation that estimates the basic reproduction number for a fully susceptible population from data on an outbreak in a population with pre-existing immunity(*S*_0_ <*N*) by correcting for this immunity using the standard SIR model assumption of homogeneous mixing. This equation reads as follows:

$$R_{0}=-\frac{N}{Y}\ln\left(1-\frac{Y}{S_{0}}\right)$$

Here *N* is the total number of hosts, *S*_0_ the total number of susceptible hosts before the outbreak (i.e. discounting from *N* any immune hosts), and *Y* the total number of susceptible hosts that became infected during the outbreak. To apply this equation, we defined a reference time interval of virus spread by identifying both a sampling point during the 2007 vector season that serves as a ‘before-outbreak’ reference as well as a sampling point close to the end of the 2007 vector season that serves as a ‘end-of-outbreak’ reference. We considered as first reference the last sampling moment during the vector season in which the number of animals that turned PCR positive and/or seropositive since the preceding sampling moment is still very small or zero. We considered as second reference that sampling point amongst those close to the end of the 2007 vector season that has the highest seroprevalence (i.e. depending the timing of the sampling moment, right before or just after the declared end of the vector season). In our default calculations we made the simplifying assumption that all replacement animals are susceptible and used a sensitivity analysis (see below) to investigate the potential influence on our results of maternal immunity in new-born animals. We set *N* equal to the total number of animals sampled at the second reference, and set *S*_0_ equal to the number of animals that were sampled at the second reference and that, if sampled at the first reference, were negative in PCR and ELISA at this first reference, plus the total number of animals PCR positive at the first reference and still present at the second reference (corresponding to individuals counted as initially susceptible and becoming infected early on in the outbreak). We set *Y* equal to the number of animals amongst the animals counted in *S*_0_ that are PCR positive and/or seropositive at the second reference; by combining PCR positivity and seropositivity here we expected to obtain the best approximation to the true prevalence of animals that have been infected by the end of the vector season. We considered the *R*_0_ values obtained in this way as lower bound estimates for the following reasons. In several herds/flocks we observed that at the second reference point both the number of PCR positive animals and the number of remaining susceptible individuals is non-zero; taking PCR positivity as an indication for infectiousness this means that it is not certain if the final outbreak size is reached as the final-size equation assumes. Further, the final size equation assumes a constant demography, which is in our case also a simplifying approximation as in all herds and flocks the composition of the population changed between the reference points. If amongst the animals that were present at the first reference but not anymore at the second there were individuals immune before the outbreak, and if all replacement animals were assumed susceptible, this approximation would also tend to underestimate the initial degree of immunity in the population during the outbreak, and thereby produce an underestimate for *R*_0_. In order to account for the potential influence on our results of maternal immunity in new-born animals, we carried out a sensitivity analysis in which we excluded from *Y* the following group of seropositive animals for which the seropositivity could be a result of maternal antibodies: all animals that underwent their first sampling at the second reference and were found seropositive but without any signal in the PCR.

### Ethics statement

No permits were required for the described study, which complied with all relevant regulations. This field study was performed in 2007/2008, two years before the release of the EU directive 2010/63 on the protection of animals used for scientific purposes. EU directive 2010/63 was implemented in Dutch law in the year 2014. No ethical license was requested at the time of this study for the involvement of animals in this study as this was considered to be part of a veterinary diagnostic procedure during an epidemic of a notifiable animal disease. For all farms, written permission to sample the animals was obtained from the owner.

## Results

### Description of prevalence patterns

**Fig 1:**
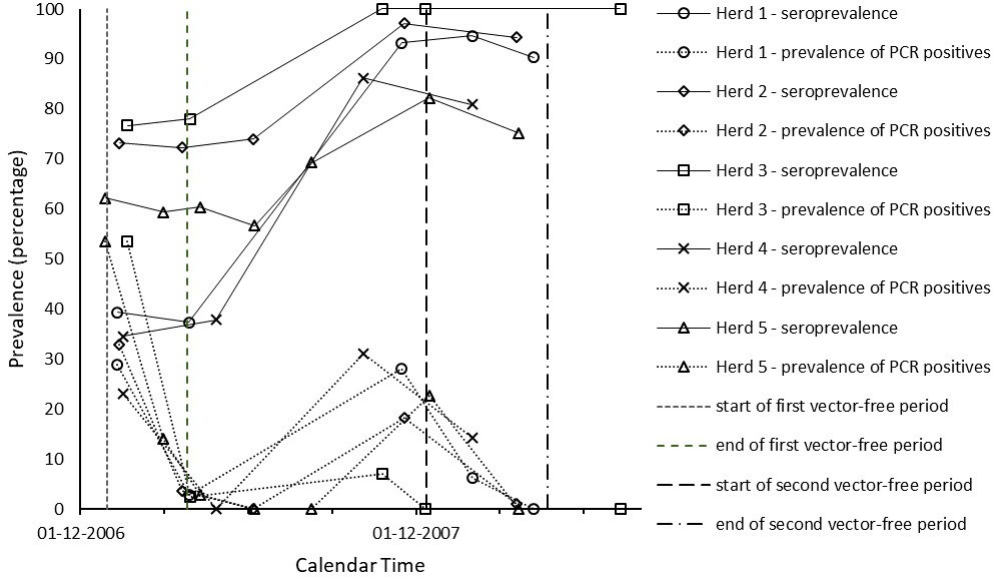
Time evolution of the percentage of test positive cattle by herd.

**Fig 2:**
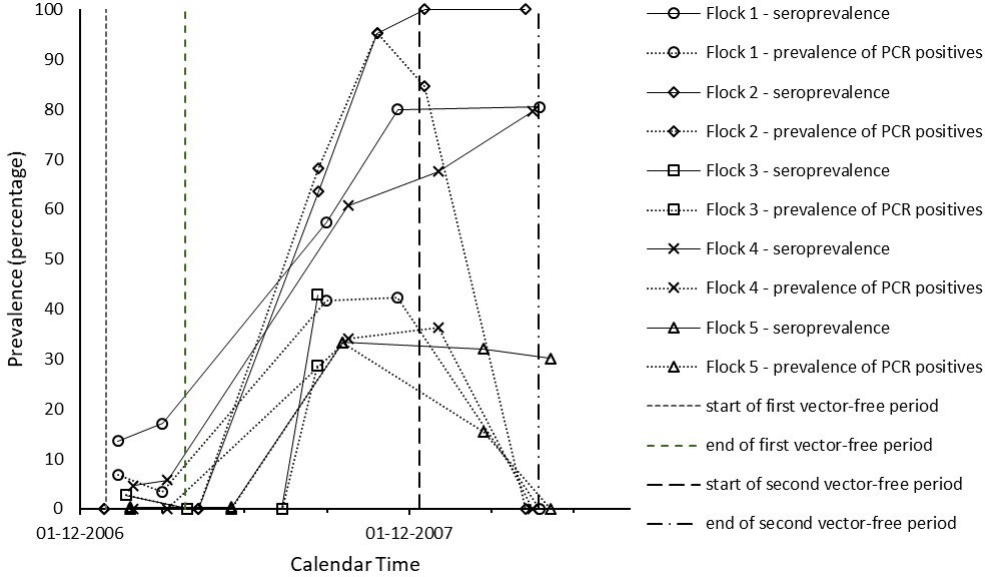
Time evolution of the percentage of test positive sheep by flock.

In Tables 1 and 2, we give a by herd/flock overview of the sampling and test results through time. The observed patterns of both seroprevalence and infection prevalence were similar across most herds/flocks, and are displayed as percentages of test positive animals in Figs 1 and 2. In all five herds and all five flocks monitored, the seroprevalence increased significantly after the start of the 2007 vector season, consistent with vector-borne virus transmission occurring in all farms monitored. Highest seroprevalence was found at sampling moments between August 2007 and January 2008, i.e. in the second half of the vector season. Virus positive animals were almost exclusively found at sampling moments in this same period, i.e. between August and January, with prevalence peaking in August-December. In the cattle herds studied, seroprevalence values were already high before the 2007 vector season and increased further during that season. Fig 1 shows that the seroprevalence at around the start of the 2007 vector season ranged between 37% (Herd 1) and 78% (Herd 3) and the maximum seroprevalence attained at around the end of this vector season ranged between 82% (Herd 5) and 100% (Herd 3). In the sheep flocks studied, the seroprevalence values were still low before the 2007 vector season and tended to increase (even) more sharply during this season than in the cattle herds. Fig 2 shows that the seroprevalence in the sheep flocks at around the start of the 2007 vector season ranged between 0% (Flock 2 and 3) and 17% (Flock 1) and the maximum seroprevalence attained at around the end of this vector season ranged between 33% (Flock 5) and 100% (Flock 2). The prevalence patterns of PCR positivity compared with the seroprevalence patterns consistently with the expectation that the duration of PCR positivity is shorter than the duration of seropositivity, and thus PCR positivity is an indicator of having been infected relatively more recently. The prevalence range of PCR positivity at around the end of the 2007 vector season (second reference point) was higher in the sheep flocks (between 29% in Flock 3 and 95% in Flock 2) than in the cattle herds (between 7% in Herd 3 to 31% in Herd 4), in line with the observed more sharp increase of seropositivity in sheep during the vector season. Conversely, in correspondence with the observation that seroprevalence in cattle was already high before the 2007 vector season, the prevalence range of PCR positivity at around the start of the first vector-free period (first reference point) was much higher in the cattle herds than in the sheep flocks studied. This agreed with the expectation that the high seroprevalence in cattle was caused relatively recently, i.e. by BTV transmission during the 2006 vector season. As can be seen from Table 1 by comparing the last column to the third, throughout the study period PCR positive cattle were all or almost all also found seropositive. In sheep, as can be seen from the same comparison in Table 2, when prevalence of PCR positivity peaked there was a minority of PCR positive animals in Flocks 1, 2 and 5 that were not (yet) seropositive.

In S1 Table we show for all herds/flocks how many animals underwent a status conversion between consecutive sampling days (animals negative in both PCR and serology turning positive in PCR and/or serology, PCR positives turning negative and seropositives turning seronegative). Negatives turned positive exclusively during the vector season as expected, except for one cow in herd 2 turning seropositive within the 2006/2007 vector-free season. Outside the vector season we observed an almost complete PCR positivity decline. We observed only a few seropositives turning negative.

### Estimation of transmission parameters

For the sampling intervals evidencing initial epidemic virus spread we estimated the net between-ruminant basic reproduction number *R*_0_. These estimates are listed in Table 3.

**Table 3:** Estimated minimum values for the within-farm basic reproduction number *R*_0_.

Herd/flock number	Reference interval	*N*	*S*_0_	*Y*	Estimated *R*_0_
Herd 1	(2,3)	103	74	67	3.6
Herd 2	(3,4)	71	30	28	6.9
Herd 4	(2,3)	29	20	16	2.9
Herd 5	(5,6)	101	54	36	3.1
Flock 1	(2,4)	90	84	66	2.1
Flock 2	(2,4)	21	21	20	3.2
Flock 3	(3,4)	14	14	8	1.5
Flock 4	(2,4)	78	73	49	1.8
Flock 5	(2,3)	432	431	164	1.3

Herd 3 was not included as throughout the study period it had very few susceptible animals such that the number of newly infected animals observed was too low for parameter inference.

## Discussion

To our knowledge this is the first report providing longitudinal field data on an ongoing BTV-8 epidemic in cattle herds and sheep flocks, and based not only on serology but also on virus antigen detection by PCR. The field data included 5 dairy herds and 5 sheep flocks and enabled a quantification of BTV within-farm spread across the full vector season of 2007. In all farms, spread of BTV infection was observed during the vector season, and in the cattle herds this spread occurred despite a substantial proportion of animals being seropositive due to BTV circulation in the previous year 2006. From the data we obtained quantitative insight into within-farm spread of BTV; in part due to the inclusion of PCR results in this study it was possible to use the final-size equation for the SIR model as a basis of the analysis, a method that requires relatively few assumptions. The transmission potential of BTV-8 within the herds and flocks studied was found to be high, particularly in the cattle herds. In the cattle herds the minimum estimates of the within-flock *R*_0_ range from 2.9 to 6.9 (one herd not analysed), which is in line, albeit with less variation, with the estimates obtained using only serological data (median *R*_0_: 2.3, 5th– 95th percentile: 1.8–11.0) by [18]. In the sheep flocks in this study the minimum estimates of the within-flock *R*_0_ range between 1.3 and 3.2. Based on the standard relationship between the critical vaccination coverage and *R*_0_, these ranges correspond to a critical vaccination coverage ranging between 66 and 85 percent for cattle and between 21 and 69 percent for sheep. The estimates are all minimum estimates due to approximations that were made in applying the final-size equation, as detailed in the Methods.

In our default calculations we ignored the possibility that replacement animals (animals that were born or bought into the population in between sampling moments) that were seropositive but not PCR positive at their first sampling could represent animals with maternal antibodies. The reason was that, as no age (nor descent) information was collected on replacement animals, we could not identify which animals were born to infected ewes or infected cows. Also, as the ELISA used cannot distinguish between IgG and IgM antibodies we could not use the presence of IgG antibodies only as an indication of maternally acquired immunity. In a sensitivity analysis we have studied the impact of assuming all animals seropositive at first sampling but without PCR signal were not infected but only carrying maternal antibodies. This led to somewhat lower minimum estimates of the within-farm *R*_0_ for three of the cattle herds and two of the sheep flocks (for details see S2 Table), resulting in *R*_0_ estimates for cattle range from 2.7 to 3.7 and the range for sheep remaining the same.

Our results for the within-flock *R*_0_ provide information for studies that estimate this parameter using more detailed models that include the vector [14–17, 25]. In such more detailed models the within-flock *R*_0_ is calculated from underlying parameters such as vector biting rate, vector preference and vector population size. Our field estimate results for the basic reproduction number of within-herd transmission in four cattle herds are consistent with range suggested in Fig 2A of Ref. [25] using literature-based plausible ranges of parameters in models including the vector. The fact that the biting rate of potential viral vector species—*C*. *chiopterus*, *C*. *obsoletus complex*, *C*. *dewulfi* and *C*. *pulicaris* [26–31] in cattle is much higher than in sheep [32, 33] would be one building block in explaining our finding that the within-herd basic reproduction number tends to be somewhat higher in cattle herds than in sheep flocks.

## Supporting information

S1 Table Number of status conversions in each sampling interval. (DOCX)

S2 Table Sensitivity analysis for the within-farm basic reproduction number. (DOCX)

S1 File Data set. (XLSX)
