# Supplementary material for: Within-farm transmission characteristics of bluetongue virus serotype 8 in cattle and sheep in the Netherlands, 2007-2008
Source: PLoS One. 2021 Feb 8;16(2):e0246565. doi: 10.1371/journal.pone.0246565 (PMC7870048; doi:10.1371/journal.pone.0246565)
Supplement: S1 Table — (DOCX) [file pone.0246565.s001.docx]

**Supporting Information** to ‘Within-farm transmission characteristics of bluetongue virus serotype 8 in cattle and sheep in the Netherlands, 2007-2008’ by Thomas J. Hagenaars et al.

**S1 Table.** Number of status conversions in each sampling interval. The sampling moments are numbered according to Table 1. Numbers in bold refer to dates that are within the vector season as defined by the official estimates of the Dutch Food Safety Authority (NVWA). ‘total_svneg’ (resp. total_vpos’ and ‘total_spos’) denotes the number of both serologically as well as PCR negative animals (resp. the number of serologically positive animals and the number of PCR positive animals) at the first sampling of the interval and that are still present at the second sampling. Amongst these animals, ‘turning pos’ (resp. ‘turning v neg’ and ‘turning s neg’) is the number of animals that turn serologically and/or PCR positive (resp. turn PCR negative and turn serologically negative) at the second sampling of the interval.

| Herd 1 | | | | | | | | | | | |  |
| --- | --- | --- | --- | --- | --- | --- | --- | --- | --- | --- | --- | --- |
| sampling interval | (1,**2**) | | (**2**,**3**) | | (**3**,4) | | (4,5) | |  |  |  |  |
| turning pos total_svneg | 0 | 59 | 53 | 53 | 2 | 7 | 0 | 6 |  |  |  |  |
| turning v neg total_vpos | 24 | 27 | 2 | 2 | 26 | 29 | 7 | 7 |  |  |  |  |
| turning s neg total_spos | 1 | 38 | 0 | 30 | 1 | 93 | 5 | 98 |  |  |  |  |
| Herd 2 | | | | | | | | | | | |  |
| sampling interval | (1,2) | | (2,**3**) | | (**3**,**4**) | | (**4**,5) | |  |  |  |  |
| turning pos total_svneg | 1 | 19 | 0 | 10 | 13 | 13 | 0 | 2 |  |  |  |  |
| turning v neg total_vpos | 19 | 21 | 1 | 1 | 0 | 0 | 12 | 12 |  |  |  |  |
| turning s neg total_spos | 2 | 54 | 1 | 33 | 0 | 27 | 1 | 55 |  |  |  |  |
| Herd 3 | | | | | | | | | | | |  |
| sampling interval | (1,**2**) | | (**2**,**3**) | | (**3**,**4**) | | (**4**,5) | |  |  |  |  |
| turning pos total_svneg | 0 | 8 | 2 | 2 | 0 | 0 | 0 | 0 |  |  |  |  |
| turning v neg total_vpos | 18 | 19 | 0 | 0 | 1 | 1 | 0 | 0 |  |  |  |  |
| turning s neg total_spos | 0 | 28 | 0 | 11 | 0 | 13 | 0 | 13 |  |  |  |  |
| Herd 4 | | | | | | | | | | | |  |
| sampling interval | (1,**2**) | | (**2**,**3**) | | (**3**,4) | |  | |  |  |  |  |
| turning pos total_svneg | 0 | 16 | 15 | 17 | 0 | 4 |  |  |  |  |  |  |
| turning v neg total_vpos | 5 | 5 | 0 | 0 | 2 | 4 |  |  |  |  |  |  |
| turning s neg total_spos | 0 | 8 | 0 | 9 | 0 | 15 |  |  |  |  |  |  |
| Herd 5 | | | | | | | | | | | | |
| sampling interval | (1,2) | | (2,**3**) | | (**3**,**4**) | | (**4**,**5**) | | (**5**,6) | | (6,7) | |
| turning pos total_svneg | 1 | 36 | 0 | 40 | 0 | 24 | 1 | 16 | 7 | 17 | 0 | 18 |
| turning v neg total_vpos | 40 | 54 | 12 | 15 | 0 | 0 | 0 | 0 | 0 | 0 | 21 | 21 |
| turning s neg total_spos | 1 | 61 | 0 | 60 | 4 | 41 | 1 | 32 | 4 | 36 | 4 | 72 |
|  |  |  |  |  |  |  |  |  |  |  |  |  |
| Flock 1 | | | | | | | | | | | |  |
| sampling interval | (1,2) | | (2,**3**) | | (**3**,**4**) | | (**4**,5) | |  |  |  |  |
| turning pos total_svneg | 0 | 61 | 43 | 46 | 5 | 19 | 2 | 15 |  |  |  |  |
| turning v neg total_vpos | 3 | 6 | 3 | 3 | 12 | 44 | 27 | 27 |  |  |  |  |
| turning s neg total_spos | 4 | 13 | 3 | 8 | 3 | 61 | 1 | 55 |  |  |  |  |
| Flock 2 | | | | | | | | | | | |  |
| sampling interval | (**2**,**3**) | | (**3**,**4**) | | (**4**,**5**) | | (**5**,6) | |  |  |  |  |
| turning pos total_svneg | 17 | 22 | 3 | 4 | 0 | 0 | 0 | 0 |  |  |  |  |
| turning v neg total_vpos | 0 | 0 | 0 | 15 | 2 | 13 | 11 | 11 |  |  |  |  |
| turning s neg total_spos | 0 | 0 | 0 | 14 | 0 | 13 | 0 | 12 |  |  |  |  |
| Flock 3 | | | | | | | | | | | |  |
| sampling interval | (1,**2**) | | (**2**,**3**) | | (**3**,4) | |  | |  |  |  |  |
| turning pos total_svneg | 0 | 14 | 0 | 14 | 7 | 13 |  |  |  |  |  |  |
| turning v neg total_vpos | 0 | 0 | 0 | 0 | 0 | 0 |  |  |  |  |  |  |
| turning s neg total_spos | 0 | 0 | 0 | 0 | 0 | 0 |  |  |  |  |  |  |
| Flock 4 | | | | | | | | | | | |  |
| sampling interval | (1,**2**) | | (**2**,**3**) | | (**3**,4) | | (4,**5**) | |  |  |  |  |
| turning pos total_svneg | 0 | 39 | 43 | 72 | 7 | 30 | 1 | 9 |  |  |  |  |
| turning v neg total_vpos | 0 | 0 | 0 | 0 | 7 | 24 | 16 | 16 |  |  |  |  |
| turning s neg total_spos | 0 | 2 | 2 | 5 | 0 | 44 | 1 | 29 |  |  |  |  |
| Flock 5 | | | | | | | | | | | |  |
| sampling interval | (1,**2**) | | (**2**,**3**) | | (**3**,4) | | (4,**5**) | |  |  |  |  |
| turning pos total_svneg | 0 | 175 | 94 | 152 | 21 | 91 | 1 | 74 |  |  |  |  |
| turning v neg total_vpos | 0 | 0 | 0 | 0 | 37 | 65 | 37 | 37 |  |  |  |  |
| turning s neg total_spos | 0 | 1 | 0 | 0 | 1 | 60 | 2 | 80 |  |  |  |  |
