# Supplementary material for: Within-farm transmission characteristics of bluetongue virus serotype 8 in cattle and sheep in the Netherlands, 2007-2008
Source: PLoS One. 2021 Feb 8;16(2):e0246565. doi: 10.1371/journal.pone.0246565 (PMC7870048; doi:10.1371/journal.pone.0246565)
Supplement: S2 Table — (DOCX) [file pone.0246565.s002.docx]

**Supporting Information** to ‘Within-farm transmission characteristics of bluetongue virus serotype 8 in cattle and sheep in the Netherlands, 2007-2008’ by Thomas J. Hagenaars et al.

**S2 Table.** Sensitivity analysis for the within-farm basic reproduction number $R_{0}$. Here we assume that all replacement animals sampled at the second reference that were seropositive but without PCR signal at their first sampling (and thus could represent animals with maternal antibodies) were not infected but only carrying maternal antibodies. Herd 3 is not included as throughout the study period it had very few susceptible animals such that the number of newly infected animals observed was too low for parameter inference.

| **Herd/flock number** | **Reference interval** | **N** | **S_0_** | **Y** | **Estimated** $\boldsymbol{R}_{\boldsymbol{0}}$ |
| --- | --- | --- | --- | --- | --- |
| Herd 1 | (2,3) | 103 | 74 | 61 | 2.9 |
| Herd 2 | (3,4) | 71 | 30 | 19 | 3.7 |
| Herd 4 | (2,3) | 29 | 20 | 16 | 2.9 |
| Herd 5 | (5,6) | 101 | 54 | 29 | 2.7 |
| Flock 1 | (2,4) | 90 | 84 | 63 | 2.0 |
| Flock 2 | (2,4) | 21 | 21 | 20 | 3.2 |
| Flock 3 | (3,4) | 14 | 14 | 8 | 1.5 |
| Flock 4 | (2,4) | 78 | 73 | 47 | 1.7 |
| Flock 5 | (2,3) | 432 | 431 | 160 | 1.3 |
